# *Solanaceae* pangenomes are coming of graphical age to bring heritability back

**DOI:** 10.1007/s42994-022-00087-0

**Published:** 2022-11-14

**Authors:** Björn Usadel

**Affiliations:** 1 Institute for Biological Data Science, CEPLAS, Heinrich Heine University, Düsseldorf, Germany; 2 IBG-4 Bioinformatics, BioSC, Forschungszentrum Jülich, Jülich, Germany

**Keywords:** Tomato, Potato, Pangenome, Graph-based pangenome, Heritability

## Abstract

Two recent articles describe a pangenome of potato and a graph-based pangenome for tomato, respectively. The latter improves our understanding of the tomato genomics architecture even further and the use of this graph-based pangenome versus a single reference dramatically improves heritability in tomato.

The last few years have seen significant progress in *Solanaceae* genomics, spurred by novel sequencing technologies. It all started with the publication of the first version of the tomato “Heinz 1706” genome 10 years ago (Tomato Genome Consortium 2012) and subsequently led to the elucidation of several *Solanaceae* reference genomes, ranging from wild to crop species. However, advances in sequencing technologies and bioinformatics have continued, enabling ever cheaper and/or better analyses techniques. This has allowed elucidating the genomes of tomato-like *Solanum* species (Molitor et al. 2021; Powell et al. 2022) which have been used in the construction of introgression lines (Chetelat et al. 2019)*.* At the same time, reduced short-read sequencing costs allowed analysing several hundred tomato accessions shedding light on domestication history (Lin et al. 2014) and ultimately led to the construction of the tomato pangenome (Gao et al. 2019). Novel long-read Nanopore data allowed the in-depth analysis of structural variations within the tomato pangenome (Alonge et al. 2020). Finally, a combination of the two competing long-read technologies (i.e. Nanopore and PacBio) showed their complementarity to construct near complete, gapless tomato assemblies (van Rengs et al. 2022). Potato genomics was following these developments closely. Here, short-read genome sequencing of a diverse panel of potato relatives, landraces and accessions shed light on the evolutionary and domestication history of potato (Hardigan et al. 2017). Long-read-based sequencing allowed the more precise reconstruction of a doubled monoploid (Pham et al. 2020) and a haplotype resolved assembly of a diploid potato in 2020 (Zhou et al. 2020). This year has already seen the release of a small, phased potato pangenome comprising six genotypes (Hoopes et al. 2022) and a novel method relying on pollen sequencing to resolve the complex autotetraploid potato genome to the four individual haplotypes (Sun et al. 2022). (Fig. 1). These resources are complemented by pepper (Ou et al. 2018) and eggplant (Barchi et al. 2021) short-read based pangenomes.

**Fig. 1:**
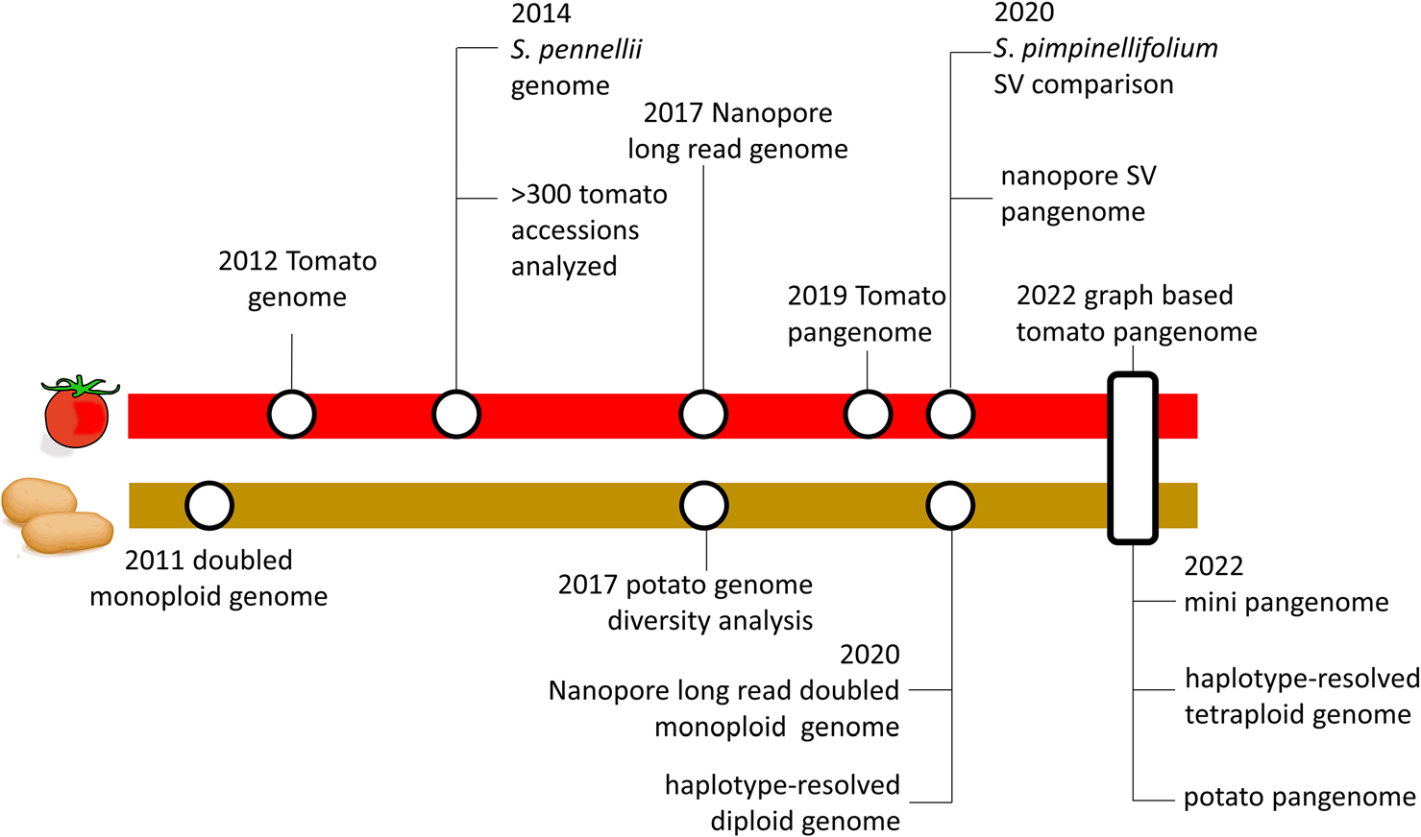
major genomics milestones for the crops tomato and potato from the genome release to reach the pangenome and graph-based pangenome stages

Two companion papers are now pushing the boundaries in *Solanaceae* genomics further by releasing novel and improved pangenomes for tomato and potato. Tang et al. (2022) explored the potato pangenome, shedding light on potato provenance and evolution, whereas Zhou et al. (2022) reconstructed the tomato pangenome using pangenome graphs integrating newly generated genome assemblies and all earlier genome data, facilitating latest developments in the pangenome field.

These are developments that will further spur genomic and genetic analysis in the *Solanaceae* family as it has become clear that a single reference genome is usually not enough to characterise and capture the genetic variation found within an entire species. Consequently, the new improved “Heinz 1706” tomato genome reference constructed by Zhou et al. (2022) comprised 36,648 protein-coding genes, whereas the addition of multiple long-read genomes allowed the identification of an additional 14,507 genes present in tomato. This new pangenome thus increased the total number of genes in the tomato clade even further compared to the short-read-based pangenome constructed earlier (Gao et al. 2019).

This is potentially partially explained by the use of the improved underlying long-read sequencing technologies, as Gao et al. (2019) argued that the tomato pangenome is likely closed, i.e., it comprises a finite total number of genes. Indeed, the novel pangenome data by Zhou also shows a tapering of new gene additions per genome as more genomes are added. Similarly, the novel potato genome also seemed to nearly reach a plateau of genes found when approximately 40 genomes were incorporated (Tang et al. 2022). In any case, focussing on the gene content allows exploring the relation of “core” genes (i.e., those that are present in all genomes) to “shell” genes that are found less often across accessions, or even those that are accession specific, or “private”. Core genes often have known functions, exhibit wider expression ranges and are usually more highly expressed, compared to dispensable genes that are more likely to have no known function and might often be on the way to pseudogenization. The remainder of non-core genes are often enriched in genes related to defence response, which has been shown for tomato (Gao et al. 2019) and has now been corroborated for potato as well. In addition, Tang et al. (2022) also observed an increased expression level of core genes compared to non-core genes. Besides these gene centric approaches, pangenomes can allow for more accurate identification of complex DNA polymorphisms than a single linear reference genome, where genomes are combined into one unifying framework. Whilst there is not yet a single standard framework or workflow to capture the pangenome unambiguously, most modern methods try to capture the genome in the structure of a graph. The novel tomato pangenome used the popular vg toolkit (Sirén et al. 2021) to represent the whole pangenome in one structure, including single nucleotide polymorphisms and structural variants. Jointly, these data were shown to be superior in calling variants from simulated genomic data and, as expected for graph-based genomes, the sensitivity to detect structural variants was markedly increased.

These new approaches have ushered tomato genomics research into the “graph”-based pangenome era. This is a necessary development as both the genome of *S. pimpinellifolium* (Wang et al. 2020), a close relative to the cultivated tomato, as well as the Nanopore-based tomato pangenome (Alonge et al. 2020) highlighted the importance of structural variations for phenotypes. Thus, it seemed mandatory to capture as much structural variation as possible for tomato.

Zhou et al. (2022) demonstrated the importance of such a graph-based pangenome by comparing the heritability of more than 20,000 molecular traits when variants were inferred using the linear genome to that when variants were derived from the graph-based pangenome. Interestingly, single nucleotide polymorphisms (SNPs) alone exhibited a slightly higher average heritability when these were derived from the pangenome. However, it was the sum of all variants that boosted average heritability to 0.41 in the graph-based pangenome, mostly driven by structural variants. This highly surpassed the estimated heritability of 0.33, which was estimated based on variants inferred from the linear genome only. This can have implications for GWAS and other studies trying to find causal genes as was demonstrated for one exemplary gene, where a structural variant (SV) led to a truncated transcript exhibiting high heritability. A statistically significantly associated SNP, however, was several genes away, which could have promoted misidentification of the most likely causal gene.

Using these novel SV data for genomic selection should, therefore, improve prediction models, which was indeed the case for the majority of 33 investigated flavour traits.

To further explore missing heritability, the authors showed that in the case of expression quantitative trait locus (eQTL) with determined *cis* regulation, considering all variants in the specific *cis* region versus only the leading eQTL increased the estimated heritability further, underlining the importance of considering allelic heterogeneity. However, as of yet, modelling all loci and allele heterogeneity cannot be efficiently incorporated into prediction models. Hence for genes, a gene co-expression network was constructed and subclusters were extracted to only use proximal genetic variation within these clustered genes. While this procedure does naturally sacrifice some heritability, it provides a useful heuristic and was shown to be particularly useful for flavonoids.

In summation, novel insights and better applicability based on increased heritability in breeding research are likely direct outcomes of the improved graph-based pangenomic tomato references.

Therefore, will this be the final tomato pangenome? Further improvements will most likely become available, as pangenomic reconstruction and representation in general is still a fast-moving field where bioinformatics and sequencing technology are still markedly improving. This is exemplified in the fact that the tomato graph-based pangenome did not specifically consider copy number variation. Furthermore, given extensive introgressions from wild species not yet included in the pangenome into breeding lines, a super pangenome (Khan et al. 2020) comprising not only close, but also more distant relatives in the tomato clade, might yield further insights. However, based on recent evidence about the assiduous *Solanum* community, this and other improvements are probably just around the corner and the myriad of applications of the graph-based tomato genome are just now becoming possible as all data is available in accessible databases.

## Data Availability

Data sharing not applicable to this article as no datasets were generated or analysed during the current study.
